# A cross-sectional survey on knowledge and perceptions of health risks associated with arsenic and mercury contamination from artisanal gold mining in Tanzania

**DOI:** 10.1186/1471-2458-13-74

**Published:** 2013-01-25

**Authors:** Elias Charles, Deborah SK Thomas, Deborah Dewey, Mark Davey, Sospatro E Ngallaba, Eveline Konje

**Affiliations:** 1 School of Public Health, Catholic University of Health and Allied Sciences, PO Box 1464, Mwanza, TANZANIA; 2 Department of Geography & Environmental Sciences, University of Colorado Denver, PO Box 173364, Denver, CO, 80217-3364, USA; 3 Departments of Paediatrics and Community Health Sciences, University of Calgary, 2888 Shaganappi Trail NW, Calgary, AB, T3B 6A8, CANADA; 4 Exploration Unit, Twigg Gold Limited, PO Box 1866, Mwanza, Tanzania

**Keywords:** Hazard risk knowledge, Perception, Artisanal mining, Arsenic and mercury, Tanzania

## Abstract

**Background:**

An estimated 0.5 to 1.5 million informal miners, of whom 30-50% are women, rely on artisanal mining for their livelihood in Tanzania. Mercury, used in the processing gold ore, and arsenic, which is a constituent of some ores, are common occupational exposures that frequently result in widespread environmental contamination. Frequently, the mining activities are conducted haphazardly without regard for environmental, occupational, or community exposure. The primary objective of this study was to assess community risk knowledge and perception of potential mercury and arsenic toxicity and/or exposure from artisanal gold mining in Rwamagasa in northwestern Tanzania.

**Methods:**

A cross-sectional survey of respondents in five sub-villages in the Rwamagasa Village located in Geita District in northwestern Tanzania near Lake Victoria was conducted. This area has a history of artisanal gold mining and many of the population continue to work as miners. Using a clustered random selection approach for recruitment, a total of 160 individuals over 18 years of age completed a structured interview.

**Results:**

The interviews revealed wide variations in knowledge and risk perceptions concerning mercury and arsenic exposure, with 40.6% (n=65) and 89.4% (n=143) not aware of the health effects of mercury and arsenic exposure respectively. Males were significantly more knowledgeable (n=59, 36.9%) than females (n=36, 22.5%) with regard to mercury (*x*^2^=3.99, p<0.05). An individual’s occupation category was associated with level of knowledge (*x*^2^=22.82, p=<0.001). Individuals involved in mining (n=63, 73.2%) were more knowledgeable about the negative health effects of mercury than individuals in other occupations. Of the few individuals (n=17, 10.6%) who knew about arsenic toxicity, the majority (n=10, 58.8%) were miners.

**Conclusions:**

The knowledge of individuals living in Rwamagasa, Tanzania, an area with a history of artisanal gold mining, varied widely with regard to the health hazards of mercury and arsenic. In these communities there was limited awareness of the threats to health associated with exposure to mercury and arsenic. This lack of knowledge, combined with minimal environmental monitoring and controlled waste management practices, highlights the need for health education, surveillance, and policy changes.

## Background

Artisanal mining is increasingly common in many parts of the world with more than 30 million active artisanal miners in more than 55 countries [1,2]. In Tanzania alone, there are an estimated 0.5 to 1.5 million informal miners, of whom 30-50% are women [1]. In fact, the number of artisanal mining sites is expanding in many regions of Tanzania, particularly around Lake Victoria and in the central and southwestern regions of the country. Artisanal mining activities are largely concentrated in rural areas that have very little infrastructure, and the individuals undertaking informal mining generally lack education, training, management skills and essential equipment for safe mining practices.

Mercury (Hg), used in the processing of gold ore, and arsenic (As), which is a constituent of some gold ores, are common occupational exposures that can result in widespread environmental contamination. Frequently, the mining activities in Tanzania are conducted haphazardly without consideration of environmental, occupational or community exposures. Further, environmental monitoring and waste management in artisanal gold mining areas in Tanzania is minimal.

Hg and As are known toxicants that are hazardous to humans, wildlife and domestic animals and may accumulate in the environment causing serious damage to ecosystems and human health [3,4]. Studies conducted throughout the world, including Tanzania, where artisanal mining occurs have reported the presence of high Hg concentrations in human urine, breast milk, blood, hair, and nails, and in plant and fish samples [5-13]. These studies have noted that Hg exposure due amalgamation and inhalation has escalated since 1972. High levels of Hg and As have been linked to detrimental effects on humans, such as skin problems, cancer, high blood pressure, cardiovascular diseases, and neurological and reproductive disorders among others [7,11]. However, the burden of disease from Hg and/or As exposure in Tanzania has not been widely examined. Other studies in Geita (Tanzania) have revealed that most of the children living in Hg-exposed areas and those working with Hg display neuropsychological deficits, which are associated with Hg exposure [5,8].

Most of the people living in close proximity to artisanal mining areas are vulnerable to Hg and As exposure. The potential harm of these toxicants to pregnant women, their fetuses and young children is an area of special concern. Women in these gold mining regions may engage in geophagy, or earth-eating, behaviors, which are common in artisanal mining areas due to poor nutritional status and cultural acceptance of this practice [14]. Modest consumption of 50 g of sikor (i.e., a moulded soil sold in the local market) from an As contaminated area per day is said to be equivalent to intake of 370 μg of As [15]; however, this depends on the degree of contamination of the soil used to make the sikor [14]. More alarming is the fact that low Hg exposure in mothers due to this practice could result in the fetus being exposed to high levels of Hg as it is concentrated by a factor of ten in the fetus relative to the mother [16]. Thus, even a woman with a low level of mercury exposure could give birth to a child with significant birth defects. In addition, Hg exposure due to transmission through breast milk could have an effect on the healthy development of infants [8]. Finally, infants and children are sometimes directly exposed to the mining processes themselves, since mothers often have their children at the mine site and processing areas while they are working and sometime young children even participate in mining as workers.

Symptoms of Hg and As exposure are characterized by peripheral neuropathy and acrodynia among others [17-20], which can result in social stigmatization among the affected individuals. Women suffer the most in this regard, and may have difficulty finding a husband as they are regarded less attractive, unhealthy and possibly as having sexual problems [21].

How a person perceives the symptoms of Hg and As poisoning and the associated morbidities and mortalities − risk perception − can affect how one acts and the decisions one makes concerning avoidance, control or protection against exposure [22]. In Tanzania, the knowledge and perception of people who live in close proximity to artisanal mining with regard to the potential health risks of exposure to Hg and As is unknown. Hg and As exposure add to the environmental burden of disease in Tanzania and could be minimizing achievement of the Millennium Development Goals for reducing child mortality, improving maternal health and ensuring environmental sustainability (i.e., MDGs 4, 5 and 7, respectively) [23]. The primary objective of this study was to assess community risk knowledge and perception of potential Hg and As toxicity and/or exposure from artisanal gold mining in Rwamagasa Village in Geita District in northwestern Tanzania.

## Methods

**Figure 1:**
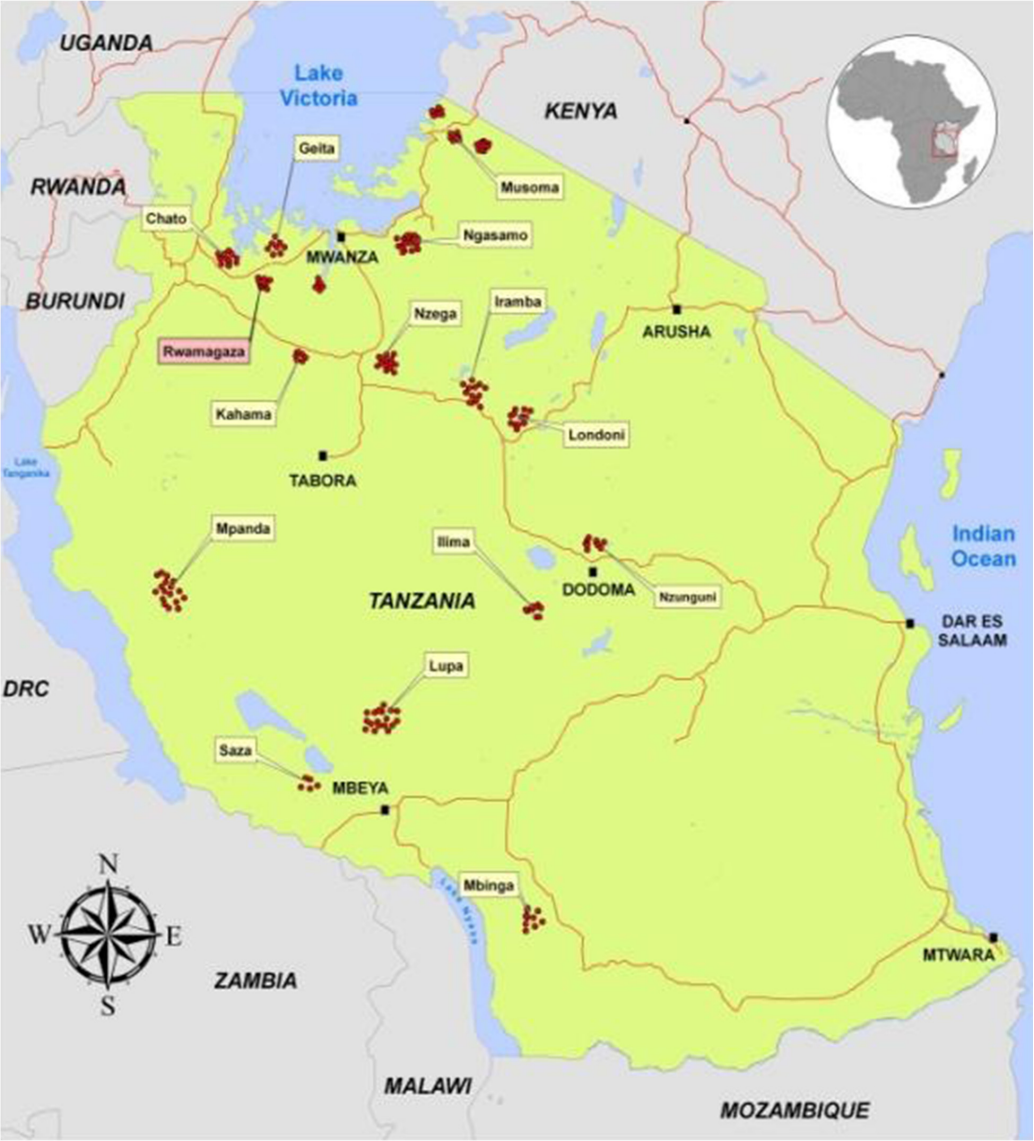
Principal artisanal mining centers in Tanzania compiled from multiple sources by authors.

The study was conducted at the village of Rwamagasa located in Geita District (7,825 km^2^) (Figure 1). Geita first came into prominence as the site of a German colonial gold mine in 1900s. Artisanal miners have been working in the area since 1972, and currently there are more than 300 active miners. The estimated total population of Rwamagasa is 7768 (3764 males and 4004 females), according to village records. Most people in the village depend on water from rivers, natural wells, and wells constructed for human and livestock consumption and for mining. Only 12% of the population has access to a protected public well [5,24,25].

Using a cross sectional design with a clustered random sampling technique, 159 people were targeted for recruitment into the study and 160 individuals consented and participated in the face-to-face structured interview. All of the individuals approached agreed to participate, (response rate was 100%). These participants were from five sub-villages (clusters), CCM (n=54), Elimu (Isenyi) (n=28), Imalanguzo (n=25), Isingilo (n=27) and Nyakayenze (n=26) with populations of 4641, 884, 603, 538, and 362 respectively. Since mining is not physically segregated from the community, but rather occurs in close proximity to housing and other economic activities, all people over the age of 18 were considered eligible to participate.

Knowledge and perception data were collected using a structured interview, which was pre-tested and amended prior to conducting fieldwork. Research assistants were recruited and were trained regarding the study’s purpose and methods to ensure consistency and reliability of data collection. In addition, the principal researcher (EC) supervised all associated activities. The survey instrument was initially designed in English, translated to Kiswahili, and then translated back to English by another translator to ensure that the translated version captured the questions correctly. The interviews were conducted in Kiswahili, which was the primary language of the interviewers and interviewees. All field data were recorded on field data sheets/checklists, and were double-entered to ensure accuracy.

The survey consisted of four sections: 1) demographics, 2) potential risk for Hg and As exposure from animal, food, and water sources, 3) knowledge and risk perception of Hg and As, and 4) mining practices, particularly with regard to environmental considerations related to Hg. The survey included categorical questions (“Yes”, “No”, “Don’t know” and “Not Applicable”), questions that required the participant to rate their response on a Likert scale (“very low”, “low”, “normal”, “high”, “very high”) to determine their level of agreement with a statement, and open-ended questions. Participant’s knowledge of Hg and As poisoning was determined from basic questions regarding their awareness of associated health impacts. Six questions were scored “1” for a correct answer and “0” for incorrect answer. Respondents were classified as knowledgeable if they obtained total scores of ≥ 2 and not knowledgeable if they obtained a total score of < 2. With respect to perceptions about the cause of the morbidities and mortalities associated with Hg and As toxicity, a similar scoring scheme was applied to questions that asked about: 1) the cause of morbidities such as skin pigmentation problems, hyperpigmentation, edema, excessive perspiration, brain damage and cancer; 2) participants agreement with the statement that Hg and As environmental contamination might be a cause of brain damage, edema, cancer, and skin pigmentation problems among individuals living in the area; and 3) whether the cause of death of a participant’s parent was associated with Hg and/or AS related symptoms. A respondent whose answer to any one of the questions indicated that they perceived Hg or As as the cause for specific health-related morbidities/mortalities was given a score of “1”. Participants who obtained a score of “1” were classified as having a clear perception; individuals with a score of less than “1” were classified as having a negative perception of Hg and As morbidities and mortalities. All questions were considered to have equal weight.

Ethical approval was obtained from the Directorate of Postgraduate Studies of the Weill Bugando University College of Health and Allied Sciences, and permission to conduct the research in Mwanza Region and Geita District was obtained from the offices of the Regional and District Commissioners. Written informed consent was obtained from each participant. Importantly and contributing to the successful response rate, the investigators obtained the support of the miners and the community for the study by visiting and introducing the purpose and importance of the study to the entire community at Rwamagasa. The investigators also provided the miners with personal protective gear, such as dust masks and latex gloves.

Data were analyzed using Statistical Package for the Social Sciences (Version SPSS-17.0) after checking and cleaning for discrepancies. Chi-square tests (Fischer’s Exact Test) were used where appropriate to determine the association between socio-demographic variables (which included sex, age, education level, marital status, economic activities and how long the person had lived in the study area) and the outcomes of interest (knowledge of potential health risks of Hg and As and associated factors were categorized into two groups: knowledgeable and not knowledgeable). The association was considered statistically significant when p-values were less than 0.05, Odd Ratios (OR) were used as a measure of association and reported with 95% confidence intervals.

## Results

The age of the participants ranged from 18 years to 71 years; however, most were between 25 and 38 years of age (n = 91, 56.9%). Forty-eight (30%) had lived in Rwamagasa for one to five years, and twenty-eight (17.5%) had lived in the area for six to ten years. Only fourteen of the respondents (n =14, 8.8%) had lived in Rwamagasa for more than 30 years. The CCM sub-village was the main center for mining activities and had the largest population. Among the study population, the proportion of participants who had not received any primary school education (referred to as illiterate) was 15.6% (n = 25) for males and 19.4% (n = 31) for females. Economic activity was defined as the major activity that the participants engaged in as the source for their secure livelihood. The majority of the sample at Rwamagasa were miners (n=86, 53.8%), followed by farmers (n=63, 39.4%). A few of the participants work in public services and retail businesses (n=11, 6.9%).

The level of knowledge about the environmental and health risks of Hg and As toxicity among individuals at Rwamagasa is summarized in Table 1. Of those surveyed, 65 individuals (40.6%) were not aware of Hg toxicity and 143 (89.4%) were not aware of As toxicity. Most of the participants (78.8%, n=126) were unable to identify any specific symptoms associated with Hg exposure. Only 21.3% (n=34) were able to identify at least one symptom. The majority of the participants (66.3%, n=106) stated there were no health effects. In comparison, when the participants were asked about malaria, most of the respondents mentioned more than one symptom, such as headache (n=36, 22.5%), body weakness (n=35, 21.9%), fever (n=29, 18.1%), profuse sweating (n=39, 21.3%) and loss of appetite (n=26, 16.3%).

**Table 1:** Knowledge about Mercury and Arsenic toxicity among individuals in Rwamagasa area

	**Group**	** Category**	**N**	**Knowledgeable**		**p-value**	**OR**	**95% CI for OR**	
				**n**	**%**			**min**	**max**
Mercury	Economic activity	Agriculture	63	23	14.4	<0.001	0.258	0.137	0.483
		Mining	86	63	39.4				
		Business & Public services	11	9	5.6				
	Education	Illiterate	56	28	17.5	0.203	0.934	0.511	1.705
		Primary	91	59	36.9				
		Secondary and above	13	8	5.0				
	Sex	Male	89	59	36.9	0.046	1.875	0.944	3.726
		Female	71	36	22.5				
Arsenic	Economic activity	Agriculture	63	2	3.2	<0.001	0.284	0.11	0.733
		Mining	86	10	11.6				
		Business & Public services	11	5	45.4				
	Education	Illiterate	56	1	1.8	0.005	0.334	0.123	0.908
		Primary	91	12	13.2				
		Secondary and above	13	4	30.8				
	Sex	Male	89	13	14.6	0.067	2.79	0.769	10.14
		Female	71	4	5.6				

Sex and the economic activity of an individual were found to be significantly associated with knowledge regarding Hg toxicity; sex (*x*^2^=3.99, p=0.046) and economic activity (*x*^2^=22.82, p<0.001). Males were significantly more knowledgeable (n=59, 36.9%) than females (n=36, 22.5%). Most of the participants (n=143, 89.4%) had no knowledge about As toxicity; however, significant associations were found between knowledge of As toxicity and economic activity (*x*^2^=17.83, p<0.001) and education level (*x*^2^=10.79, p=0.005). This indicates that, of the few individuals (n=17, 10.6%) who knew about As toxicity, most were miners (n=10, 58.8%) and most (n=16, 94.1%) had primary education and above.

There was significant variability among respondents in terms of their perceptions regarding the cause of the morbidities and mortalities associated with Hg and As toxicity. Some of the respondents associated morbidities related to Hg and As exposure with an evil spirit or witchcraft (n=33, 20.6%), others associated them with god’s curse (n=17, 10.6%), and still others indicated that they were caused by contagious diseases, such as HIV/AIDS (n = 64, 40%). Only 46 individuals (28.8%) associated the Hg and As morbidities and mortalities with Hg and As toxicity (Table 2).

**Table 2:** Perception on mercury and arsenic toxicity and related morbidities and mortalities

**Variables/Features**	**Category**	**Frequency(n)**	**%**
Specific perception on Mercury and Arsenic toxicity and related morbidities and mortalities^a^	An evil spirit or Witchcraft	33	20.6
	God’s curse	17	10.6
	Contagious disease	64	40.0
	Chemical poisoning	46	28.8
Agreement with Mercury and Arsenic toxicity^b^	Strongly agree	46	28.8
	Uncertain (not sure)	78	48.8
	Disagree	30	18.8
	Strongly Disagree	6	3.8
Cause of parental death among occupants^c^	Mercury and arsenic related symptoms	10	28.6
	Chest problems	9	25.7
	Superstition and assassinations	11	34.3
	Other diseases	5	11.4
Perception status on Mercury and Arsenic morbidities and associated mortalities	Clear perception	50	31.2
	Negative perception	110	68.8

## Discussion

In Rwamagasa, along with the high proportion of people earning their livelihood from mining, the low literacy levels of the random sample of individuals who participated in this study suggest that risk reduction through education, training, behavioral modifications, land use decisions, and health care delivery could be challenging. However, a lesson on improving the livelihood of artisanal miners can be taken from Sadiola in western Mali, where a successful diversification of artisanal miners was achieved in 1997 by introducing alternative means for securing livelihood [26]. Fundamentally, the environmental health of the community must be addressed without jeopardizing the rights of individuals to secure a livelihood.

Even with the increase in artisanal mining activities in Tanzania, community-based and/or occupational health education programs that focus on the health hazards of Hg and/or As exposure are severely lacking. In Tanzania, the amount of money and effort spent on the risks education of miners is considerably lower than that spent on enforcement and monitoring [27,28]. In Rwamagasa for instance, there was only one poster advocating the use of cleaner technology located at the ward executive’s office. This likely had little effect because of the low level of literacy of the individuals who visit the office. Health promotion campaigns on the recognition of the symptoms and signs of Hg and As poisoning should be established in rural communities with artisanal mining, including Rwamagasa. Further, local health facilities should be equipped and health workers trained to conduct heavy metal testing [29].

Even with knowledge and awareness, there is often no relationship between how one acts and the decisions one makes concerning avoidance, control or protection against exposure [22,28]. So, while the findings of this study indicated that the majority of the miners had some basic knowledge about Hg toxicity and associated morbidities, it has been reported that artisanal miners continued to use Hg with their bare hands and to burn the amalgam in open air [24,27]. The necessity of generating a livelihood often outweighs the potential negative health outcomes.

Women and children carry a particular burden with regard to toxic exposures. Even though women are usually the primary caregivers to children, they are less likely to know about the health effects associated with Hg exposure compared to men and so are disadvantaged in making decisions about exposure reduction. Additionally, at every mining location visited by the researchers, women miners were observed with their infants and young children. Children were also often directly involved in working directly in the mining activities. Thus, not only is the mother directly exposed to Hg, but so are her children.

Among the respondents, almost half reported knowing about abnormalities, including birth defects and skin discoloration, among infants in the study area. Although not all birth defects are caused by toxic chemicals, birth defects are more common in areas where industries, such as artisanal gold mining use or produce toxic chemicals or wastes such as Hg [30]. Further research is needed that examines the relationships among Hg and As exposure and congenital anomalies in this population.

Some signs of Hg poisoning are easy to confuse with malaria [17,30,31], which has implications for awareness and subsequent prevention and treatment. The following symptoms are associated with both malaria and Hg poisoning: peripheral neuropathy, itching, hypotonia (muscle weakness), tachycardia, nausea and sometimes vomiting, visual problems, profuse sweating, headache, respiratory tract infections, fever, rigors, tiredness, myalgia (limbs and back), abdominal pain, loss of appetite, hypertension (especially for Hg poisoning) and postural hypotension (especially in malaria), enlarged liver and spleen, coma and eventually death [17,31]. Malaria is a common chronic health problem in this region [32], and so people generally have a relatively high degree of awareness and familiarity with it. In addition to the questions about Hg, respondents were also asked about malaria symptoms; perhaps not surprisingly, most participants were able to correctly identify at least one symptom of malaria, but were not able to do the same for Hg poisoning. There is a need for further investigation on the co-morbidities of chronic Hg exposure with malaria in artisanal mining areas.

Partnership is absolutely essential in addressing the elements of environmental health and in reducing the health risks due to artisanal mining, as noted in the Ottawa Charter for Health Promotions [22]. Governments, donors, NGOs and other stakeholders should emphasize the necessity of recognizing the importance of artisanal mining and focus on creating supportive environments for building social-economic capital, including legal, financial, technical, cultural, and political issues [27]. This could provide individuals and groups with access to resources and support that reduce the health risks to those involved in artisanal mining and the surrounding communities, as well as reducing the impact of artisanal mining on the environment.

## Conclusions

A majority of participant in this study had limited awareness and knowledge about the health risks associated with Hg and As exposures, irrespective of economic activities or educational level. However, there was significant variability among the various groups. Overall, miners were more knowledgeable than individuals in other occupations, and men had a higher degree of awareness than women. In fact, a majority of miners identified Hg as a health hazard. The impact of As exposure was less known by the participants. At a minimum, based on the findings of this study, it is recommended that a health promotion campaign be established in Rwamagasa Village, Tanzania that addresses the health hazards associated with Hg and As. The overall lack of knowledge, combined with minimal environmental monitoring and controlled waste management practices, highlights the need for health education, surveillance, and policy changes. Future research that examines the pathways of contamination, exposure, and the burden of disease is also needed.

## Competing interests

The authors declare that they have no competing interests. It should be noted that Twigg Gold is not engaged or associated with artisanal mining in the Rwamagasa belt or anywhere else.

## Authors’ contributions

EC designed the study, developed the survey, supervised the data collection, analyzed the data and wrote the paper. DSKT contributed to the study design and development of the survey, the interpretation of the findings, as well as the drafting and writing of the manuscript. DD contributed to the development of the overall study design and interpretation of results. MD contributed to the study design and survey. SEN contributed to the study design, survey, and analysis/interpretation of the data. EK contributed to the study design and data analysis. All authors read and approved the final manuscript.

## Pre-publication history

The pre-publication history for this paper can be accessed here:

http://www.biomedcentral.com/1471-2458/13/74/prepub
